# Properties of untranslated regions of the *S. cerevisiae *genome

**DOI:** 10.1186/1471-2164-10-391

**Published:** 2009-08-22

**Authors:** Tamir Tuller, Eytan Ruppin, Martin Kupiec

**Affiliations:** 1 School of Computer Science, Tel Aviv University, Ramat Aviv 69978, Israel; 2 Department of Molecular Microbiology and Biotechnology, Tel Aviv University, Ramat Aviv 69978, Israel; 3 School of School of Medicine, Tel Aviv University, Ramat Aviv 69978, Israel

## Abstract

**Background:**

During evolution selection forces such as changing environments shape the architecture of genomes. The distribution of genes along chromosomes and the length of intragenic regions are basic genomic features known to play a major role in the regulation of gene transcription and translation.

**Results:**

In this work we perform the first large scale analysis of the length distribution of untranslated regions (promoters, 5' and 3' untranslated regions, terminators) in the genome of the yeast *Saccharomyces cerevisiae*. Our analysis shows that the length of each open reading frame (ORF) and that of its associated regulatory and untranslated regions significantly correlate with each other. Moreover, significant correlations with other features related to gene expression and evolution (number of regulating transcription factors, mRNA and protein abundance, evolutionary rate, etc) were observed. Furthermore, the function of genes seems to have an important role in the evolution of these lengths. Notably, genes that are related to RNA metabolism tend to have shorter untranslated regions and thus tend to be closer to their neighbouring genes while genes coding for cell wall proteins tend to be isolated in the genome.

**Conclusion:**

These results indicate that genome architecture has a significant role in regulating gene expression, and in shaping the characteristics and functionality of proteins.

## Background

The distribution pattern of genes throughout the genome is of utmost importance: As each gene has to be expressed under very specific circumstances and at a very specific level, genes should be isolated from each other such that their expression does not interfere with the regulation of adjacent genes. Cis-acting sequences (commonly termed *promoter *sequences) are usually located 5' to the transcriptional initiation site of each gene. Binding of transcription factors and chromatin modifiers at these sites allows appropriate gene expression [1]. However, it is to be expected that the traverse of RNA polymerase, a large multi-protein complex of high molecular weight, through an upstream gene, may interfere with the binding of these regulators. Genes that are divergently expressed (i.e. share a promoter) usually share transcription factors, and show similar regulation. Thus, many times such genes are functionally related. Interestingly, convergent genes, in which two RNA polymerases could potentially collide, do not usually exhibit transcriptional interference [2,3], due to the presence of sequences that act as transcriptional *terminators*, acting on both strands [4].

Most mRNAs in *S. cerevisiae *are typically about 300 nucleotides longer than their translated sequences [5]. The untranslated regions at the 5' (5'UTRs) and at the 3' (3'UTRs) of genes seem to play important roles in gene regulation. For example, it was found that 5'UTRs and 3'UTRs include conserved stem-loop structures that are involved in the coordinated post-transcriptional regulation of biological pathways [6]. 5'UTRs have been implicated mainly in translational control, affecting all post-transcriptional stages, including mRNA stability, folding, and interactions with the ribosomal machinery [7-14]. In addition, it was found that 3'UTRs have important roles in mRNA stability [15,16] and localization [17]. It has also been suggested that a minimal distance between genes in *S. cerevisiae *is required for successful transcription. The observed distances between genes have been shown to fit such a theoretical model of gene distribution [18,19]. These results imply additional constraints on the lengths of untranslated regions. Previous studies have shown that ORF length significantly correlates with features such as their expression levels [20,21]. However, it is not clear if similar connections (possibly with other features) can be found when considering the lengths of untranslated regions.

**Figure 1:**
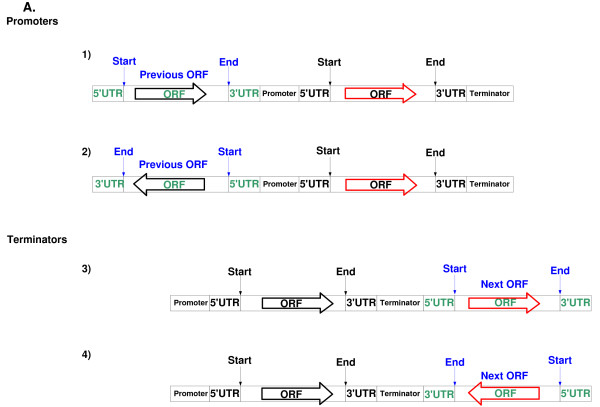
**Schematic representation of the definition of Promoters, 5'UTRs, 3'UTRs, and terminators**. Two types of promoters appear in parts 1) and 2) Two types of terminators appear in parts 3) and 4). Note that that in cases 1) and 3) the terminator of one gene is the promoter of the next gene. Thus, in the case of terminators, we treated this category separately from the converging case [4].

The first paper that analyzed gene distribution in *S. cerevisiae *appeared shortly after the genome sequence was released [22]. Recently, a large-scale measurement of the lengths of UTRs in *S. cerevisiae *was performed [23,24]. These data enable us to accurately estimate the lengths of the untranslated regions of thousands of *S. cerevisiae *genes. Using these length estimations we perform the first large scale analysis of length distributions of coding and non coding regions in the yeast genome. We aim at improving our understanding of the determinants that are related to the length of each non-coding region (promoter, 5'UTR, 3'UTR, terminator; exact definitions are given in the next section; see Figure 1), and learning about the relation between length distribution of non-coding regions and the functionality of the corresponding genes.

## Results and discussion

In order to gain initial information about the organization of functionally related genes in the genome, we measured, for each open reading frame (ORF), the distance (in nucleotides) to its neighboring ORFs, and asked whether genes with similar functional roles or characteristics (i.e., genes sharing GO annotations) tend to be closer to other genes or isolated (see Additional file 1). Table 1 (left) identifies GO groups whose genes tend to be closer-than-average to their neighbouring genes. These are highly enriched for categories related to RNA metabolism (splicing, RNA binding proteins, etc.). In contrast, the GO groups that tend to be isolated from other genes (Table 1, right) show enrichment for cell wall proteins (glucanases, proteins that promote flocculation, etc.), plasma membrane proteins, and transmembrane sugar transporters. All these categories share the property that the proteins encoded by these genes are located at the cell periphery, either at the membrane or the cell wall. The fact that very specific categories are enriched implies that the tendency of genes to be isolated or not in the genome has a clear functional value.

**Table 1:** GO groups whose genes tend to be close to neighbouring genes (left) and GO groups that tend to be isolated from other genes (right).

**GO groups whose genes tend to be close to neighbouring genes**	**GO groups that tend to be isolated from other genes**
**nuclear mRNA splicing, via spliceosome (p < 0.001)**	**plasma membrane (p < 0.001)**

**retrotransposon nucleocapsid (p < 0.001)**	**chitin- and beta-glucan-containing cell wall (p < 0.001)**

**RNA binding (p < 0.001)**	flocculation via cell wall protein-carbohydrate interaction (p < 0.001)

**Protein binding (p < 0.001)**	glucose transmembrane transporter activity (p = 0.001)

**transposition, RNA-mediated (p < 0.001)**	DNA helicase activity (p = 0.002)

**telomere maintenance (p = 0.001)**	fructose transmembrane transporter activity (p = 0.002)

DNA-directed DNA polymerase activity (p < 0.001)	mannose transmembrane transporter activity (p = 0.002)

RNA-directed DNA polymerase activity (p < 0.001)	telomere maintenance via recombination (p = 0.003)

ribonuclease activity (p < 0.001)	helicase activity (p = 0.003)

Spliceosome (p < 0.001)	hexose transport (p = 0.003)

peptidase activity (p < 0.001)	endonuclease activity(p = 0.004)

RNA splicing factor activity, transesterification mechanism (p < 0.001)	---

U4/U6 × U5 tri-snRNP complex (p < 0.001)	---

Group I intron splicing (p = 0.001)	---

tRNA methylation (p = 0.002)	---

peroxisomal membrane (p = 0.003)	---

DNA-dependent DNA replication (p = 0.004)	---

tRNA splicing (p = 0.004)	---

mRNA catabolic process (p = 0.004)	---

Cytokinesis (p = 0.005)	---

snRNP U1 (p = 0.005)	---

Corresponding p-values appear in brackets. GO groups whose p-values are significant after FDR correction (Materials and methods) are in bold.

See Additional file 1 for more results and p-values and the Materials and methods for details about how these p-values were computed.

**Figure 2:**
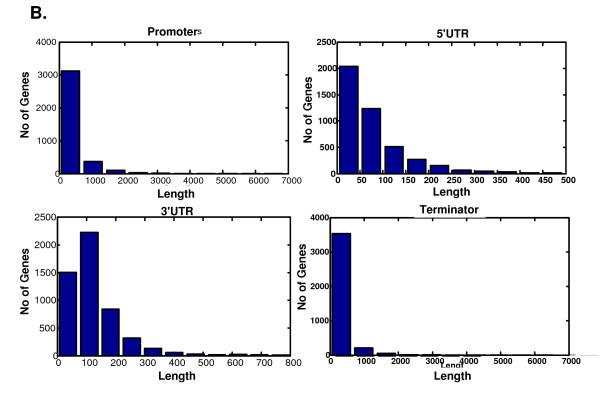
**Length distribution of untranslated regions**.

The results presented were obtained by measuring the distance between the beginning of the gene's ORF and the end of the previous ORF, and similarly, from the end of the gene's ORF to the beginning of the ORF in the subsequent gene. Thus, this first characterization ignores gene orientation and the site of transcription initiation/termination. Recently, the precise transcription initiation and termination sites have been determined in a genome-wide fashion [23,24]. This allows us to define, for each gene, the length of the regions that are transcribed but not translated: 5' and 3'UTRs (Figure 1). We thus divide the yeast genome into the following categories: For two genes transcribed in the same direction, we define the promoter of the downstream gene to be the region between the 3'UTR end of the upstream gene and the beginning of the 5'UTR of the gene in question. This region should also contain, at the same time, signals required to terminate transcription of the upstream gene. However, it has been shown that most of the signals for 3' mRNA generation are within the transcribed region [25]. Thus, one can adjudicate to most of these sequences a role as transcription regulators of the downstream gene. In the case of divergently expressed genes, these usually share a promoter region (defined as the distance between the beginning of the two 5'UTRs). In the case of converging genes, these share a terminator, that contains cis-acting sequences that prevent transcriptional collision between incoming RNA polymerases [4] (Figure 1). We measured the size of all genes and intergenic regions in the yeast genome. Additional file 2 includes the length of promoters, 5' UTRs, ORFs, 3' UTRs and terminators of all the *S. cerevisiae *genes for which this information was available. The length distribution of untranslated regions appears in Figure 2. As can be seen, each of these distributions has a single peak with an average of 455, 83, 136, and 275 bp for the promoters, 5'UTRs, 3'UTRs, and terminator correspondingly. The standard deviations of these distributions are in the same order of magnitude; 919, 84, 138, and 765 correspondingly.

## Functional distribution of genes

To study the functional significance of the differences in size observed, we computed the length of the various intergenic regions for each GO group. The average length of each of the gene parts for each GO category was calculated, and compared to the rest of the genome. Additional file 3 includes p-values (for being longer or shorter than average) for the lengths of the promoters, terminators and UTRs of each GO functional category.

Table 2 summarizes the cellular functions (Biological Process ontology) that have extremely long or short promoters/terminators/UTRs. Consistent with the results presented in Table 1, GO groups related to RNA metabolism (transcription, splicing, RNA binding) display short promoters. Interestingly, genes involved in the response to DNA damage (DNA repair, DNA damage response, homologous recombination) can also be placed in this category (Table 2A). rRNA processing and ribosome components are highly enriched among 5'UTRs that are shorter than average. Ribosomal proteins also tended to have shorter than average 3'UTR. The short UTRs of ribosomal proteins may facilitate their regulation as part of the Environmental Stress Response (ESR) [26].

**Table 2:** Summary of the cellular functions (Biological Process ontology) with extreme promoters, UTRs and terminators in *S. cerevisiae*.

**Short Promoter**	**Short 5'UTR**	**Short 3'UTR**	**Short Terminator**	**Short End to End Terminator**
1. Response to DNA damage stimulus	1. rRNA processing 2. Protein folding	**---**	**---**	**---**
2. DNA repair				
3. nuclear mRNA splicing, via spliceosome				
4. protein transport				
5. RNA elongation from RNA polymerase II promoter				
6. RNA splicing				
7. mRNA processing				
8. chromatin modification				
9. DNA recombination				

**Long Promoter**	**Long 5'UTR**	**Long 3'UTR**	**Long Terminator**	**Long End to End Terminator**

**--**	**1**. protein amino acid phosphorylation	1. regulation of transcripti on, DNA-dependent	**1**. response to stress **2**. amino acid transport	--
	**2 **. signal transduction			
	**3**. cell wall organization and biogenesis			
	**4**. pseudohyphal growth			
	**5**. endocytosis			
	**6**. metabolic process			
	**7**. small GTPase mediated signal transduction			
	**8**. invasive growth.			

All the p-values corresponding to the GO groups presented in this table are lower than 0.0048. The length distribution of all functional categories is presented in Additional file 3, technical details appear in the Materials and Methods section.

No particular GO group exhibited longer than expected promoters (Table 2B). This suggests that the GO groups found in Table 1 to be isolated from their neighbouring genes, such as cell wall and plasma membrane proteins, do not require this distance to accommodate larger promoters where more transcription factors can bind (see below). In contrast to the lack of larger-than-average promoters, many GO groups were enriched for long 5'UTRs. These included categories related to signal transduction pathways (amino acid phosphorylation, signal transduction, small GTPase signal transduction), invasive and pseudohyphal growth, and cell wall proteins. Long 5'UTRs have been linked in the past to translation regulation: folding of the 5'UTR may help regulate the accessibility to the ribosome [8]. Indeed, all the processes mentioned require precise levels of expression. Our results suggest that they may be regulated at the level of initiation of translation. Table 2B also shows that genes involved in transcription regulation tend to have long 3'UTRs (probably pointing to regulation through RNA binding proteins, see below), whereas longer than usual terminators can be seen in genes involved in response to stress and amino acid transport (Table 2B). The length distribution of all functional categories is presented in Additional File 3.

Next, we asked whether there is a correlation between the length of the different regions of each gene. Table 3 shows that the highest correlations are seen between the size of each ORF and its 5' UTR (a correlation of 0.19), as well as between the promoter and terminator regions (0.16). These results may suggest that longer genes require longer regulatory regions. Indeed, such genes are regulated on average by more transcription factors (correlation = 0.12, p < 10^-16^; see the next section) and their mRNA tend to bind more regulatory proteins (correlation = 0.16, p < 10^-16^; see the next section); these features may require longer promoters and UTRs (see the next section). Interestingly, the adjacent 3'UTR and terminator regions exhibit a clear and strong negative correlation (-0.19). The opposing trends between 3'UTR and its adjacent terminator region suggest that a minimal distance must exist between ORFs to allow proper expression levels. This results in a trade-off between the 3'UTR length and that of the terminator [18].

**Table 3:** Spearman correlations (and p-values) between the lengths of Promoters, UTR5s, UTR3s, and Terminator.

	ORF	Promoter	5' UTR	3' UTR	Terminator
ORF	--------	**0.053 (0.0015)**	**0.19** **(< 10^-16^)**	-0.032 (0.02)	**0.073** (5.66*10^-6^)

Promoter	--------	---------	0.0717 (1.37*10^-5^)	**0.043** **(0.01)**	**0.155** (1.37*10^-16^)

5' UTR	--------	---------	---------	0.1 (4.63*10^-11^)	**0.124** (1.37*10^-12^)

3' UTR	--------	---------	---------	---------	**-0.19 (< 10^-16^)**

Terminator	--------	---------	---------	---------	---------

Significant correlations after FDR correction are shown in bold.

### Factors related to the length of the different regions

In the next stage, we analyzed whether the different gene regions are correlated with different factors that affect gene expression. The following variables were analyzed (Table 4): 1) Number of transcription factors known to bind at the promoter region (N° of TFs) [27]. 2) Number of RNA binding proteins known to bind its mRNA product (N° of RPB) [28]. 3) mRNA levels [29]. 4) mRNA half life [30]. 5) 5'UTR free energy [8]. 6) Protein abundance (PA) [31]. 7) Protein half life [32]. 8) Noise in protein levels [33]. And 9) Evolutionary rate of the gene (ER) [34]. In the case of variables with small discrete number of values (N° of TFs, N° of RBF), the correlation is reported as significant only when an empirical p-value corresponding to a permutation test was significant (see Materials and methods; the empirical p-values appear in Additional File 4).

**Table 4:** Relations between the lengths of Promoters, 5'UTRs, 3'UTRs, and various parameters.

	**ORF**	**Promoter**	**5'UTR**	**3'UTR**	**Terminator**
**No of TFs**	**0.12**	**0.29**	**0.13**	0.099	**0.15**

**No of RBP**	**0.16**	**0.05**	**-0.066**	**0.091**	0.034

**mRNA levels**	**-0.139**	**0.062**	**-0.107**	**0.043**	0.039

**mRNA half life**	**0.12**	0.01	**0.08 **	**-0.065 **	0.036

**5' free Energy **	-0.02	**0.063**	**0.059**	**0.057**	0.035

**PA noise**	-0.034	**0.116 **	0.051	0.031	-0.011

**PA**	**-0.147**	**0.127**	**-0.099**	0.036	**0.065**

**Protein half life**	**-0.271**	-0.0012	**-0.135 **	-0.02	-0.028

**ER**	0.049	**-0.08 **	-0.018	**-0.051**	-0.044

We correlations that are significant (both empirical p-value < 0.01 and p-value < 0.01, and FDR correction) are bolded. The p-values appear in Additional file 4.

Table 4 shows that the length of ORFs and untranslated regions significantly correlate with many central features. For example, as expected, a positive correlation can be seen between promoter length and the number of transcription factors binding it (r = 0.29, p < 10^-16^). However, the fact that the number of TFs also correlates with terminator and 5'UTR lengths additionally suggests that genes with more extensive TFs regulation require longer distance from neighboring ORFs.

Genes with higher protein abundance and increased mRNA levels tend to have longer promoters, UTR3, and terminators, and tend to be short (presumably, to allow efficient translation; see for example [35]). This result demonstrates that the untranslated regions contribute to the tighter regulation of highly expressed genes. In addition, proteins whose abundance within the cell tends to be variable or "noisy" show longer promoters. The significance of this observation remains unclear.

Interestingly, we found a significant negative correlation between promoter length and evolutionary rate of the corresponding genes. This correlation is still significant after controlling for the number of TFs or for any of the other features that appear in Table 4. Thus genes with longer promoters evolve at a slower rate. This seems to occur independently of the fact that they are regulated by more TFs, and tend to have higher mRNA and protein levels. The puzzling inverse correlation between promoter length and evolutionary rate suggests that regulatory mechanisms other than TFs play an important regulatory role, which cannot be easily modified during evolution. This additional regulatory mechanism(s) could be related to chromatin configuration, an aspect of nuclear architecture that has lately been the focus of much attention [36].

Throughout the years various roles have been attributed to the 5' and 3' UTR regions, including mRNA stability, folding, interactions with the nuclear export, RNA processing, splicing and translational machines, as well as intracellular traffic and localization [6-17]. We show that whereas the 3' UTR length exhibits a negative correlation with mRNA half life, the 5' UTR length is inversely proportional to protein half life and abundance (Table 4). These results show that the main effect that these two untranslated regions have on gene expression occurs at two different levels, the 3'UTR acting mainly at the RNA stability level, and the 5'UTR enabling appropriate translation. Lately it has become apparent that RNA-binding proteins (RBPs) play an important role in regulating gene expression [28]. RBPs recognize specific sequences at various locations along the mRNA molecule. Our results suggest that those at the 3'UTR play a major role in regulation, as the correlation of the number of RBPs is significantly positive with the length of the 3'UTRs (0.092, p = 3.6*10^-11^) and significantly negative with the length of the 5'UTRs (-0.066, p = 1.3*10^-5^).

The organization of genomes is a subject of intensive research. Not long ago, it was assumed that genes were randomly distributed in eukaryotic genomes, in contrast to prokaryotes, where the organization of genes in regulatory operons requires their physical clustering [37]. However, work carried out in the last few years has challenged this view (reviewed in [38]). It appears that gene distribution is far from random and many eukaryotic genomes include clusters of genes that are related in their function [39,40]. A clear connection was found between co-expression and proximity, as closely-located genes tend to be co-expressed [41,42], clusters of co-expressed genes in mammalian genomes are evolutionarily conserved [42,43], and highly expressed genes and housekeeping genes tend to cluster [44-47]. In addition, clustered genes tend to exhibit similar functionality [39,40,48-50], tend to be located in domains with low recombination rates [51], encode proteins that tend to interact physically [38,52,53], and belong to the same metabolic pathway [54-56].

A number of previous publications explored the genomic distribution of genes belonging to the same biological function or biochemical pathway [48,50,55]. Recently, Tuller *et al*. compared the genomes of 16 organisms and found a high level of functional organization for eukaryotes, such as *Saccharomyces cerevisiae *[57]. They also found that the genomic distribution of cellular functions tends to be more similar in organisms that have higher evolutionary proximity. Here we analyze the distribution of genes in the genome of the yeast *Saccharomyces cerevisiae *from a functional point of view. Measuring distances between genes belonging to various GO categories, we find that certain functions in yeast are encoded by genes that tend to be close to other genes (not necessarily from the same function). We see an enrichment of functions related to mRNA splicing (Table 1). Such a clustering is explained by the fact that these genes tend to have short promoters (Table 2). The biological significance of this finding is not completely clear. One possibility is that for unknown reasons, genes related to mRNA splicing tend to be regulated by fewer transcription factors than others, and thus require shorter promoter regions. Although these genes have a lower number of transcription factors, the difference with the rest of the genome is not statistically significant (data not shown), suggesting that additional forces may affect promoter length of these genes. Alternatively, proper regulation of this set of genes may require physical proximity between transcription initiation factors and upstream regulators such as transcription factors and chromatin remodelers. Interestingly, chromatin remodelers by themselves constitute another GO group with short promoters. Additional GO groups with short promoters include those related to genome maintenance (DNA repair, DNA damage response, etc). In contrast, GO groups involved in responses to environmental changes (signal transduction, cell wall, etc.) tend to have longer untranslated sequences.

Our results suggest that gene distribution in the genome has evolved to allow suitable regulation: highly expressed genes tend to be shorter, and have extensive promoters and terminators. The longer promoters can partially be explained by the need of tighter regulation of these genes by TFs; the longer terminator may be needed in order to reduce transcription noise from neighbor genes. In addition we have shown that 5' and 3' UTRs may provide additional layers of regulation, with 3'UTRs exerting their effect at the RNA level, and 5'UTRs affecting translation levels. Thus, genome architecture has a significant role in regulating gene expression, and in shaping the characteristics and functionality of proteins.

## Conclusion

We conclude that there is significant relation between the genomic organization of untranslated regions (promoters, 5' and 3' untranslated regions, and terminators) and features of the corresponding proteins (e.g. functionality, expression levels, expression noise and evolutionary rate).

## Materials and methods

### Various Sources of Data

Information about the GO annotation and gene-order in *S. cerevisiae *was downloaded from NCBI. The GO ontology network was downloaded from OBO Foundry Ontologies . The information about gene lengths was downloaded from Biomart [58]. We used the genetic interaction network data from [59]. ChIP-chip information of 203 TFs was downloaded from the work of Harbison *et al*. [27]. We considered only interactions with *p-value *≤ .0.001. The *S. cerevisiae *gene evolutionary rates were downloaded from [34]. The protein abundance of *S. cerevisiae *in YEPD was downloaded from the work of [31]. The measurements of the half life time of *S. cerevisiae *mRNAs was downloaded from [30]; we removed negative values (very stable mRNAs). We averaged all the half life measurements of each gene; we also analyzed mRNA half life of [29] and got similar results. The measurements of protein half life were downloaded from [32].

The information about the targets of 40 RNA-Binding Proteins was downloaded from the work of Hogan et al. [28]. We considered only interactions with *q-value *≤ 0.05.

The information about the folding free energies of the most strongly folded structure of 5'-UTRs was downloaded from [8]. We considered the free energy that is related to (5'-UTR 100 nt) which is very close to the average length of the 5'UTR (83 nt, see Figure 2). mRNA levels were downloaded from [29]; we also analyzed mRNA levels of [60] and got similar results. Noise of protein abundance was downloaded from [33]; we used the DM values in YEPD.

### The lengths of the Promoters, 5'UTRs, and 3'UTRs and Terminators of *S. cerevisiae *genes

Data with the lengths of gene 5'UTRs, and 3'UTRs were downloaded from [23] (which is more complete than the data of [24] and [61]). These data were used for computing the length of promoters and terminators of genes when applicable (*i.e*. when all the information was available). See Figure 1 for the two definitions of promoters, and the two definitions of terminators.

Additional File 2 includes the lengths of UTRs, promoters, and terminators that were used in this study; missing cells denote cases where the information was not available (for UTRs) or when the information was not enough to compute the corresponding values (for terminators or promoters). The table includes 6605 ORFs; we had the information of the length of the 5'UTRs of 4420 genes, the length of 3668 promoters, the length of 5213 3'UTRs, and the length of 3849 terminators (2102 of them are convergent).

### P-values and correlations

#### GO Groups with Genes that Tend to be Far or Close to other Genes

In this test we computed for each GO group the average distance of gene in the group from the closest gene (not necessarily from the group). We generated 1000 random permutations of the genes locations and recomputed this average. Finally, for each GO group, we computed two empirical p-values (fraction of permutations where the GO group has lower or equal average distance, and with higher or equal average distance) that reflect the tendency of a GO group to be close/far from other genes.

In this case, we checked separately all the GO groups (Additional file 1, first sheet), and the largest GO groups (we used a cut-off of 60 genes to get the top largest GO groups; Additional file 1, second sheet). In the first case, due to the large number of GO groups and the fact that the smallest empirical p-value is 0.001 no GO group passed the FDR test. In the second case, several GO groups passed the FDR test.

#### P-values and correlations

We used Kolmogorov-Smirnov test to compare the distributions of the lengths of the 5'UTRs, 3'UTRs, and promoters of GO groups to the distribution in the entire genome. We considered only the largest GO groups (we used a cut-off of 35, 25, and 20 genes for Biological Processes, Molecular Functions, and Cellular Components respectively to get the top largest GO groups in the corresponding ontologies). These p-values underwent FDR correction. The results for the three ontologies appear in Additional file 3.

Some of the analyzed parameters had small discrete numbers of values (*e.g*.: number of TFs or RBP). In such cases, the standard Spearman correlation p-values are biased (they are more significant than they should be). Thus, we also computed empirical p-values by comparing the correlation to the correlations after permuting the vectors.

#### FDR

P-values were filtered by False Discovery Rate (FDR) to correct for multiple testing [62]. More specifically, first, all the p-values were sorted in increasing order, *P*_1_, *P*_2_, .., *P*_*n*_. Next, we filtered p-values, .

## Authors' contributions

TT, MK and ER participated in the design of the study; TT performed all the analysis; TT and MK participated in the preparation of this manuscript.

## Supplementary Material

Additional file 1 **Table S1**. P-value for being close or far from other genes for each GO group. Click here for file

Additional file 2 **Table S2**. Length for each ORF, Promoter, 5'UTR, 3'UTR, and Terminator. Click here for file

Additional file 3 **Table S3**. For each GO group, p-values for having long/short Promoters, 5'UTRs, 3'UTRs, and Terminators. Click here for file

Additional file 4 **Table S4**. P-values and empirical p-values for the spearman correlations between the lengths of the Promoters, UTR5s, UTR3s, and various parameters. Click here for file
