# Supplementary material for: Properties of untranslated regions of the S. cerevisiae genome
Source: BMC Genomics. 2009 Aug 22;10:391. doi: 10.1186/1471-2164-10-391 (PMC2737003; doi:10.1186/1471-2164-10-391)
Supplement: Additional file 4 — Table S4. P-values and empirical p-values for the spearman correlations between the lengths of the Promoters, UTR5s, UTR3s, and various parameters. [file 1471-2164-10-391-S4.doc]

**A.**

**Empirical p-values (Methods) for the Spearman correlations between the lengths of the Promoters/UTR5s/ORFs/UTR3s/Terminators and various parameters with small discrete number of values.**

**B.**

**P-values for the Spearman correlations between the lengths of the Promoters/UTR5s/ORFs/UTR3s/Terminators and various parameters.**
